# Datasets of small intestine and systemic immune components, transcriptomics in jejunal tissue, fecal 16S rRNA gene amplicon sequences, and fecal and plasma metabolome, in young female and male progeny dietary exposed to food additive E171 from preconception

**DOI:** 10.1016/j.dib.2026.113380

**Published:** 2026-11-02

**Authors:** Mohammad Issa, Chloé Michaudel, Blanche Guillon, Marine Guinot, Marta Grauso, Jérôme Lecardonnel, Luc Jouneau, Céline Chapuis, Hervé Bernard, Stéphane Hazebrouck, Florence Castelli, François Fenaille, Eric Gaultier, Gilles Rivière, Eric Houdeau, Karine Adel-Patient

**Affiliations:** a Université Paris-Saclay, CEA, INRAE, UMR Département Médicaments et Technologies pour la Santé (DMTS)/SPI/Laboratoire d’Immuno-Allergie Alimentaire, 91191, Gif-sur-Yvette, France; b Université Paris‐Saclay, INRAE, AgroParisTech, GABI, Jouy‐en‐Josas, France; c Université Paris‐Saclay, INRAE, UVSQ, VIM, 78350, Jouy‐en‐Josas, France; d Université Paris-Saclay, CEA, INRAE, UMR Département Médicaments et Technologies pour la Santé (DMTS)/SPI/Laboratoire innovations en spectrométrie de masse pour la santé, MetaboHUB, 91191, Gif-sur-Yvette, France; e Toxalim UMR1331 (Research Centre in Food Toxicology), Toulouse University, INRAE, ENVT, INP-Purpan, UPS, Toulouse, France; f Agence nationale de sécurité sanitaire de l’alimentation, de l’environnement et du travail (ANSES), Direction de l’Evaluation des Risques, 14 rue Pierre & Marie Curie, Maisons-Alfort, France

**Keywords:** Immunotoxicity, Chronic early life exposure, Mice, Food additive, Microbiome, Metabolome, Transcriptome, Flow cytometry

## Abstract

We recently demonstrated in mice that exposure from gestation to early post-weaning windows to a common food additive, food-grade titanium dioxide (E171), increased the susceptibility to food allergy of the young male progeny – but not that of females. Moreover, we evidenced alteration in intestinal homeostasis at baseline in males. To go further in analyzing sex-related effect, this article describes multi-omics datasets obtained from both male and female young progeny exposed to E171 and from control mice.

BALB/cAnNRj future dams were exposed to E171 from pre-pregnancy to the end of lactation (post-natal day 20, PNDD20) through E171-enriched (10 mg/kg of body weight/day) or control pellets. Then, the progeny was exposed to the same diet as their mother until euthanasia on PND30. Various samples were obtained from female and male progenies in order to assess intestinal and systemic homeostasis in exposed versus control young mice. The article includes the protocols used to process samples for the different analysis and the multi-omics datasets obtained: i) raw flow cytometry data (.fcs) obtained on cells extracted from lamina propria, Peyer’s patches and spleen, ii) concentrations of Th1/Th2/Th17 cytokines and of total IgGs, IgA and IgM antibodies in plasma, iii) raw 16S rRNA gene amplicon sequencing data obtained from feces, iv) peak intensities and characteristics of the metabolites annotated through unsupervised metabolomics analysis of plasma and feces, and v) raw transcriptomics data obtained on jejunum tissue. Each dataset is available in the INRAE data repository (https://entrepot.recherche.data.gouv.fr/dataverse/inrae) with respective DOI: MICROBIOTA: doi:10.57745/CNCI1D, METABOLITES: doi:10.57745/OUEPQM, IMMUNE COMPONENTS: Flow cytometry data, cytokines and antibody concentrations: doi:10.57745/VUDUMU; TRANSCRIPTOMICS: doi:10.57745/JYWYGV.

These unique multi-omic datasets provide valuable resources for further investigating the molecular mechanisms underlying E171 immunotoxicity, and its associated biomarkers. Datasets can be reused to compare with other experimental protocols assessing toxicity of food additives. Data from non-exposed mice can also be re-use on its own as a comparator for inter-laboratory analysis in young male and female BALB/cAnNRj mice. We would like to mention that additional organs were collected at euthanasia from male and female progeny (brain, liver, kidney, reproductive tracts, lungs) and can be provided upon request to authors.

Specifications Table

**Table:** 

Subject	Health Sciences, Medical Sciences & Pharmacology
Specific subject area	Assessment of immunotoxicity of food additives during the pre and perinatal period.
Type of data	Immune components: raw flow cytometry data of immunophenotyping performed on lamina propria, Peyer’s patches and spleen (.fcs files), and concentrations of Th1/Th2/Th17 cytokines and of total IgG, IgA and IgM in plasma (.xlsx file) 16S rRNA gene amplicon sequencing: Raw data (fastq files) Metabolomics: intensities and characteristics of annotated metabolites obtained from plasma and feces thanks to liquid chromatography coupled with high resolution mass spectrometry (LC—HRMS) and specific informatic workflow (.xlsx files) Transcriptomics on jejunum tissue: raw data (.txt files and microarray pictures)
Data collection	Samples were collected from experimental mice on PND27 (fecal microbiota) and at euthanasia (PND30; blood, feces, intestine and spleen). Cytokines (IFNγ, IL-5, IL-6, IL-10, IL-13, IL-17 and TNFα) and antibodies (IgG1, IgG2a, IgG2b, IgG3, IgA and IgM) in plasma were assayed using multiplexed commercial immunoassays (xMAP® Luminex technology and BioPlex®200 apparatus). Cells were extracted from spleen, lamina propria and Peyer’s patches and labeled to assess frequency of various innate and adaptive immune cells through flow cytometry using a Attune NxT apparatus. Preparation of purified DNA libraries and sequencing of amplicons were carried out using MiSeq Illumina technology (@Bridge platform) LC-HRMS was performed on an Ultimate 3000 chromatographic system coupled to a Q-Exactive mass spectrometer fitted with an electrospray source operating in the positive and negative ionization mode (MetaboHub platform). RNA was extracted from mid-jejunum tissue, and transcriptomic analysis was performed by hybridization on Mouse G3 gene expression v2 8 × 60 K microarrays (@Bridge platform).
Data source location	Fastq files and transcriptomics data were collected in Jouy-en-Josas (INRAE, France), flow cytometry, metabolomics and cytokines/antibodies were collected in Saclay (Université Paris-Saclay/CEA/INRAE, France)
Data accessibility	Immune components datasets Repository name: data.gouv.fr/dataverse/inrae Data identification number: doi:10.57745/VUDUMU Direct URL to data: https://entrepot.recherche.data.gouv.fr/dataset.xhtml?persistentId=doi:10.57745/VUDUMU 16S rRNA amplicon sequencing datasets Repository name: data.gouv.fr/dataverse/inrae Data identification number: doi:10.57745/CNCI1D Direct URL to data: https://entrepot.recherche.data.gouv.fr/dataset.xhtml?persistentId=doi:10.57745/CNCI1D Metabolomic datasets Repository name: data.gouv.fr/dataverse/inrae Data identification number: doi:10.57745/OUEPQM Direct URL to data: https://entrepot.recherche.data.gouv.fr/dataset.xhtml?persistentId=doi:10.57745/OUEPQM Transcriptomic datasets Repository name: data.gouv.fr/dataverse/inrae Data identification number: doi:10.57745/JYWYGV Direct URL to data: https://entrepot.recherche.data.gouv.fr/dataset.xhtml?persistentId=doi:10.57745/JYWYGV
Related research article	Issa M, Michaudel C, Guinot M, Grauso-Culetto M, Guillon B, Lecardonnel J, Jouneau L, Chapuis C, Bernard H, Hazebrouck S, Castelli F, Fenaille F, Gaultier E, Rivière G, Houdeau E, Adel-Patient K. Long-term exposure from perinatal life to food-grade TiO(2) alters intestinal homeostasis and predisposes to food allergy in young mice. Allergy. 2024;79(2):471–484. doi:10.1111/all.15960

## 1 Value of the Data

• These data provide information on local and systemic immune system, intestinal tissue, fecal microbiota, and fecal and systemic metabolome in young male and female BALB/cAnNRj mice exposed or not to E171 from pre-conception.
• Due to the need for limiting animal experiments, and taking into account provided limitations, these data can be reused as comparators in the context of toxicity assessment of other food additives or contaminants
• Multi-omics data from control mice can be used for studies such as inter-lab comparisons of BABL/c mice parameters at baseline.

## 2 Background

Intestinal homeostasis results from complex interactions between the intestinal epithelial barrier, the gut-associated immune tissue (GALT), and the gut microbiota. Its establishment starts *in utero* and amplifies at birth under the stimulation of antigens from the environment, notably those derived from diet and microbiota. Many factors, including environmental chemical stressors, can disrupt this process, thus contributing to the increased susceptibility to immune-related disorders such as food allergy later in life.

We recently evidenced in BALB/cAnRj mice that young male progeny exposed from pre-conception to the food additive E171 (TiO_2_) incorporated into pellets at a dose comparable to human exposure (10 mg/kg body weight/day) showed altered oral tolerance induction and enhanced food allergy reaction to cow’s milk proteins compared to non-exposed animals. Surprisingly, these effects were not observed in female progeny. At baseline (10 days post-weaning, postnatal day 30 – PND30), we evidenced altered intestinal homeostasis with both increased intestinal permeability, microbiota dysbiosis and altered immune system maturation in young male [1].

The present data paper aims at sharing multi-omics datasets obtained from both male and female young progenies exposed or not to E171 during gestation, lactation and early post-weaning windows.

## 3 Data Description

The data are divided into 4 datasets named “IMMUNE COMPONENTS”, “MICROBIOTA”, “METABOLITES”, and “TRANSCRIPTOME”, each containing raw and/or analyzed data folders.

The “IMMUNE COMPONENTS” dataset is untitled “Datasets of small intestine and systemic immune components in BALB/cAnRj young female and male mice exposed or not to food additive E171 from preconception”. In this dataset, the “Issa_Cytometry_raw_data.zip” file contains individual mice raw data (.fcs) of immunophenotyping of Peyer’s patches (PP), lamina propria (LP) and spleen (SPL) performed on PND30. Data were obtained using 3 different flow cytometry panels aiming at analyzing various immune populations. The file contains also the FMO (Fluorescence Minus One) raw data used for the gating strategy, and the raw data used for establishing the compensation matrices. The “Issa_Cytokines_Antibodies” file (.XLSX) provided the concentrations of IFNγ, IL-5, IL-6, IL-10, IL-13, IL-17 and TNFα, and of total IgG1, IgG2a, IgG2b, IgG3, IgM and IgA antibodies assayed in plasma from individual mice on PND30. Data were obtained thanks to xMAP®Luminex technology (commercial kits, BioPlex®200 apparatus and software).

The “MICROBIOTA” dataset is untitled “Datasets of fecal microbiota in young BALB/cAnRj female and male mice exposed or not to food additive E171 from preconception”. In this dataset, the Issa_Microbiota_Raw_Data.zip file contains all 16S rRNA amplicon sequencing raw data as fastq files for extraction blanks and mice fecal samples collected at PND27. 16S rRNA amplicon sequencing includes forward (R1) and reverse (R2) reads (“R1_XXX.fastq” and “R2_XXX.fastq”).

The "METABOLITES" dataset is entitled “Datasets of fecal and plasma metabolites in young BALB/cAnRj female and male mice exposed or not to food additive E171 from preconception”. The untargeted metabolomic analysis were performed using liquid chromatography coupled to high resolution mass spectrometry (LC-HRMS). To have a comprehensive view of metabolites, LC-HRMS was performed following two LC acquisition modes (HILIC and C18), associated with negative and positive ionization mode, respectively. Issa_annotated_metabolites_plasma.xlsx and Issa_annotated_metabolites_feces.xlsx files contain peak intensities of annotated metabolites detected in each of the samples. The files also describe the acquisition characteristics (LC-HRMS mode, m/z, retention time), confidence of annotation, chemical class and KEGG identifier of each annotated metabolite.

The “TRANSCRIPTOMICS” dataset is untitled “Datasets of jejunal transcriptome in young BALB/cAnRj female and male mice exposed or not to food additive E171 from preconception”. In this dataset, the transcripts detected in mid jejunum tissue from individual mice on PND30 are provided as .txt documents. Those data are provided in “Results” file, also containing QC reports (.pdf) for each of the six Mouse G3 gene expression v2 8 × 60 K microarrays slides used in the analysis. All quality controls with associated plots and QC reports, and scanned images are provided in Control-Qualite_R and Images files, respectively.

## 4 Experimental Design, Materials and Methods

### 4.1 Experimental design

BALB/cAnNRj mice (center d’Elevage René Janvier CERJ, Le Genest Saint-Isle, France) were housed in filtered cages under normal pathogen-free husbandry conditions, in a temperature-controlled environment, and with a strict 12 h light/dark cycle. Four-week-old future dams were acclimated for two weeks before experimentation. Three days before starting the experiments, they were randomly allocated to cages corresponding to the experimental groups. Future dams received a diet based on standard powder (LASQCdiet® Rod16-R, Genobios, Laval, France; 16.9% of proteins; deprived of animal proteins) in which commercial E171 (previously characterized [2]) was incorporated (E171 exposed group) or not (control group) (INRAE/SAAJ facilities, Jouy-en-Josas, France). The incorporation was calculated to provide a dose comparable to human exposure, i.e., 10 mg E171/kg of body weight/day ([3] and EFSA scenario, 2021 - https://doi.org/10.2903/j.efsa.2021.6585). Future dams were exposed to E171/control pellets for 30 days (preconception phase) before mating with unexposed 8 to 10-week-old males, and the exposure was pursued during gestation and lactation. At weaning (i.e.*,* PND20), male and female progenies were separated and received the same pellets as their mothers. To avoid any litter-effect, litters were randomly dispatched to different subgroups, consisting of 6–8 mice per cage descending from different mothers. Progeny groups were constituted by n = 44 for E171-exposed females, n = 29 for E171-exposed males, n = 32 for control females, and n = 32 for control males.

At PND27, feces samples were individually collected from all mice to assess microbiota composition. At PND30, blood (100 µl) and feces were collected from all mice to assess cytokines/antibodies concentrations and the plasma and fecal metabolome, respectively. A subgroup of mice was euthanised and various organs were collected on PND30 for cellular flow cytometry and transcriptomics analysis (“basal” subgroup, n = 8 /exposure and sex group – one male from control group, #24, died on day 28 so data are not available). For these 31 mice (number 1 to 32), it is to note that blood was collected by retro-orbital punction from anesthetized mice, whereas all other mice were subjected to sub-mandibular punction – these later mice were subjected from PND30 to oral tolerance and food allergy induction protocols, as described in Issa et al. [1]. All samples were treated and analyzed individually, and mouse and samples identification is provided Table 1. For some few samples, metabolomic data could not be obtained due to insufficient sample volume or quantity (mentioned as “NA” in Table 1).

**Table 1:** Mouse and sample identification.

Mouse number	sex (F = 0/M = 1)	Exposure (control=0/ E171=1)	Basal(0) / no basal(1)	Sample name for metabolites - Feces	Sample name for metabolites - Plasma	Sample name for cytokines	Sample Name for ileal transcriptome	Sample Name for 16S Sequencing in feces	Sample name for cytometry (Peyer's Patches & spleen)
1	0	0	0	1	1	1	257480917338_202212011145_S01_GE1_1200_Jun14_1_3.txt	FECES 39	9
2	0	0	0	2	2	2	257480917338_202212011145_S01_GE1_1200_Jun14_2_4.txt	FECES4	10
3	0	0	0	3	3	3	257480917339_202212011208_S01_GE1_1200_Jun14_2_3.txt	FECES 173	11
4	0	0	0	4	4	4	257480917340_202212021221_S01_GE1_1200_Jun14_2_2.txt	FECES 40	12
5	0	0	0	5	5	5	257480917341_202212021244_S01_GE1_1200_Jun14_1_4.txt	FECES3	13
6	0	0	0	6	6	6	257480917342_202212021244_S01_GE1_1200_Jun14_2_2.txt	FECES 38	14
7	0	0	0	7	7	7	257480917343_202212021244_S01_GE1_1200_Jun14_2_2.txt	FECES1	15
8	0	0	0	8	8	8	257480917340_202212021221_S01_GE1_1200_Jun14_1_3.txt	FECES2	16
9	0	1	0	9	9	9	257480917338_202212011145_S01_GE1_1200_Jun14_1_4.txt	FECES 141	41
10	0	1	0	10	10	10	257480917339_202212011208_S01_GE1_1200_Jun14_2_1.txt	FECES 69	42
11	0	1	0	11	11	11	257480917340_202212021221_S01_GE1_1200_Jun14_2_3.txt	FECES 68	43
12	0	1	0	12	12	12	257480917341_202212021244_S01_GE1_1200_Jun14_2_1.txt	FECES 70	44
13	0	1	0	13	13	13	257480917343_202212021244_S01_GE1_1200_Jun14_1_4.txt	FECES 203	45
14	0	1	0	14	14	14	257480917340_202212021221_S01_GE1_1200_Jun14_1_4.txt	FECES 142	46
15	0	1	0	15	15	15	257480917341_202212021244_S01_GE1_1200_Jun14_2_4.txt	FECES 204	47
16	0	1	0	16	16	16	257480917342_202212021244_S01_GE1_1200_Jun14_2_1.txt	FECES 140	48
17	1	0	0	17	17	17	257480917338_202212011145_S01_GE1_1200_Jun14_1_1.txt	FECES10	1
18	1	0	0	18	18	18	257480917338_202212011145_S01_GE1_1200_Jun14_2_2.txt	FECES 184	2
19	1	0	0	19	19	19	257480917339_202212011208_S01_GE1_1200_Jun14_1_2.txt	FECES9	3
20	1	0	0	20	20	20	257480917340_202212021221_S01_GE1_1200_Jun14_1_1.txt	FECES6	4
21	1	0	0	21	21	21	257480917341_202212021244_S01_GE1_1200_Jun14_2_3.txt	FECES11	5
22	1	0	0	22	22	22	257480917342_202212021244_S01_GE1_1200_Jun14_1_1.txt	FECES8	6
23	1	0	0	23	23	23	257480917343_202212021244_S01_GE1_1200_Jun14_1_1.txt	FECES7	7
24	1	0	0	NA	NA	NA	NA	NA	NA
25	1	1	0	25	25	25	257480917338_202212011145_S01_GE1_1200_Jun14_2_3.txt	FECES 33	33
26	1	1	0	26	26	26	257480917339_202212011208_S01_GE1_1200_Jun14_1_4.txt	FECES 32	34
27	1	1	0	27	27	27	257480917342_202212021244_S01_GE1_1200_Jun14_1_3.txt	FECES 79	35
28	1	1	0	28	28	28	257480917342_202212021244_S01_GE1_1200_Jun14_2_4.txt	FECES 31	36
29	1	1	0	29	29	29	257480917340_202212021221_S01_GE1_1200_Jun14_1_2.txt	FECES 30	37
30	1	1	0	30	30	30	257480917341_202212021244_S01_GE1_1200_Jun14_1_2.txt	FECES 209	38
31	1	1	0	31	31	31	257480917342_202212021244_S01_GE1_1200_Jun14_2_3.txt	FECES 34	39
32	1	1	0	32	32	32	257480917343_202212021244_S01_GE1_1200_Jun14_2_3.txt	FECES 163	40
33	0	0	1	33	33	33		FECES 183	
34	0	0	1	34	34	34		FECES5	
35	0	0	1	35	35	35		FECES 42	
36	0	0	1	36	36	36		FECES 181	
37	0	0	1	37	37	37		FECES 180	
38	0	0	1	38	38	38		FECES 99	
39	0	0	1	39	39	39		FECES 182	
40	0	0	1	40	40	40		FECES 98	
41	0	0	1	41	41	41		FECES 176	
42	0	0	1	42	42	42		FECES 175	
43	0	0	1	43	43	43		FECES 177	
44	0	0	1	44	44	44		FECES 91	
45	0	0	1	45	45	45		FECES 89	
46	0	0	1	46	46	46		FECES 41	
47	0	0	1	47	47	47		FECES 90	
48	0	0	1	48	48	48		FECES 174	
49	0	0	1	49	49	49		FECES 179	
50	0	0	1	50	50	50		FECES 96	
51	0	0	1	51	51	51		FECES 94	
52	0	0	1	52	52	52		FECES 178	
53	0	0	1	53	53	53		FECES 97	
54	0	0	1	54	54	54		FECES 93	
55	0	0	1	55	55	55		FECES 92	
56	0	0	1	56	56	56		FECES 95	
57	0	1	1	57	57	57		FECES 74	
58	0	1	1	58	58	58		FECES 29	
59	0	1	1	59	59	59		FECES 162	
60	0	1	1	60	60	60		FECES 75	
61	0	1	1	61	61	61		FECES 160	
62	0	1	1	62	62	62		FECES 28	
63	0	1	1	63	63	63		FECES 76	
64	0	1	1	64	64	64		FECES 78	
65	0	1	1	65	65	65		FECES 208	
66	0	1	1	66	66	66		FECES 161	
67	0	1	1	67	67	67		FECES 73	
68	0	1	1	68	68	68		FECES 77	
69	0	1	1	69	69	69		FECES 26	
70	0	1	1	70	70	70		FECES 147	
71	0	1	1	71	71	71		FECES 151	
72	0	1	1	72	72	72		FECES 146	
73	0	1	1	73	73	73		FECES 149	
74	0	1	1	74	74	74		FECES 144	
75	0	1	1	75	75	75		FECES 148	
76	0	1	1	76	76	76		FECES 152	
77	0	1	1	77	77	77		FECES 150	
78	0	1	1	78	78	78		FECES 145	
79	0	1	1	79	79	79		FECES 143	
80	0	1	1	80	80	80		FECES 71	
81	0	1	1	81	81	81		FECES 155	
82	0	1	1	82	82	82		FECES 153	
83	0	1	1	83	83	83		FECES 156	
84	0	1	1	84	84	84		FECES 154	
85	0	1	1	85	85	85		FECES 158	
86	0	1	1	86	86	86		FECES 159	
87	0	1	1	87	87	87		FECES 206	
88	0	1	1	88	88	88		FECES 207	
89	0	1	1	89	89	89		FECES 157	
90	0	1	1	90	90	90		FECES 205	
91	0	1	1	91	91	91		FECES 72	
92	0	1	1	92	92	92		FECES 27	
93	1	0	1	93	93	93		FECES 111	
94	1	0	1	94	94	94		FECES 44	
95	1	0	1	95	95	95		FECES 112	
96	1	0	1	96	96	96		FECES 45	
97	1	0	1	97	97	97		FECES 110	
98	1	0	1	98	98	98		FECES 187	
99	1	0	1	99	99	99		FECES 188	
100	1	0	1	100	100	100		FECES 185	
101	1	0	1	101	101	101		FECES 103	
102	1	0	1	102	NA	102		FECES 100	
103	1	0	1	103	103	103		FECES 101	
104	1	0	1	104	104	104		FECES 43	
105	1	0	1	NA	105	105		FECES 102	
106	1	0	1	106	106	106		FECES 104	
107	1	0	1	107	107	107		FECES 107	
108	1	0	1	108	108	108		FECES12	
109	1	0	1	109	109	109		FECES 106	
110	1	0	1	110	110	110		FECES 108	
111	1	0	1	111	111	111		FECES 109	
112	1	0	1	112	112	112		FECES 186	
113	1	0	1	113	113	113		FECES 105	
114	1	1	1	114	114	114		FECES 35	
115	1	1	1	115	115	115		FECES 172	
116	1	1	1	116	116	116		FECES 87	
117	1	1	1	117	117	117		FECES 37	
118	1	1	1	118	118	118		FECES 85	
119	1	1	1	119	119	119		FECES 36	
120	1	1	1	120	120	120		FECES 86	
121	1	1	1	121	121	121		FECES 88	
122	1	1	1	122	122	122		FECES 166	
123	1	1	1	123	123	123		FECES 164	
124	1	1	1	124	124	124		FECES 82	
125	1	1	1	125	125	125		FECES 165	
126	1	1	1	126	126	126		FECES 167	
127	1	1	1	127	NA	127		FECES 210	
128	1	1	1	128	128	128		FECES 81	
129	1	1	1	129	129	129		FECES 80	
130	1	1	1	130	130	130		FECES 84	
131	1	1	1	131	131	131		FECES 83	
132	1	1	1	132	132	132		FECES 168	
133	1	1	1	133	133	133		FECES 171	
134	1	1	1	134	134	134		FECES 170	
135	1	1	1	135	135	135		FECES 169	
136	1	1	1	136	136	136		FECES 212	
137	1	1	1	137	137	137		FECES 211	

Mice were identified individually, and all samples were analyzed separately. The table provides the mouse ID, sex (female = 0, male = 1), exposure group (control = 0, E171 = 1), and whether the mouse was euthanised on day 30 for sample collection for cellular and transcriptomic analyses (basal = 0) or was instead subjected to food allergy / oral tolerance induction (no basal = 1). The table also lists the identifiers of the corresponding files available in the data repositories, including data on fecal and plasma metabolites, plasma cytokines, the ileal transcriptome, fecal microbiota, and flow cytometry analyses of cells isolated from Peyer’s patches (PP), lamina propria (LP) and the spleen.

NA: Not available because the mouse died before the scheduled euthanasia.

Empty cells: Analyses not performed (corresponding to mice coded as 1 in the Basal/no basal column).".

We would like to mention that additional organs were collected at euthanasia from male and female progeny (brain, liver, kidney, reproductive tracts, lungs) and can be provided upon request to authors.

### 4.2 Assessment of systemic and intestinal immunity

#### 4.2.1 Assessment of systemic and intestinal cellular immunity

Immediately after euthanasia, the small intestine and the spleen were collected and kept at + 4 °C.

Small intestine was flushed with 10 ml of ice-cold phosphate buffered saline (PBS). After collection of a small intestine section (5 mm cuts, mid-jejunum) for transcriptomics analysis (see below), Peyer's patches (PP) were collected and cell suspension was prepared by manual dissociation through a cell strainer (70 μm; pluriSelect Life Science, Leipzig, Germany). Cells were washed in PBS with 2 mM EDTA and 2% FCS, centrifuged (600 x g, 6 min at 4 °C), and then kept in FACS buffer (PBS, 2 mM EDTA, 2% FCS, 0.1% of 1 µg/mL azide, and 20 µg/mL DNase I) on ice until labeling.

After PP removal, the intestine was longitudinally opened and cut into small pieces (about 1 cm long). Cells of interest were extracted from lamina propria (LP) following a protocol adapted from Ferrer-Font et al. [4]. First, epithelial cells were removed by three sequential incubations in calcium and magnesium-free HBSS, 2 mM EDTA. Then, extracellular matrix was digested (RPMI, containing 20% fetal calf serum (FCS), 0.222 U/mL Collagenase A (Sigma-Aldrich), and 145 Kuntz U/mL DNase I (Sigma-Aldrich) for 30 min at 37 °C while shaking at 200 rpm on an orbital shaker and vortexing every 5 min. The digested tissues then the resulting supernatants were pressed through a 100 µm and 40 µm cell strainers, respectively. Cells were washed in PBS with 2 mM EDTA and 2% FCS, centrifuged (600 x g, 6 min at 4 °C), and then kept in FACS buffer on ice until labeling.

The spleen was mechanically dissociated using a Gentle MACs dissociator and associated C-tubes (Miltenyi Biotec GmbH, Bergisch Gladbach, Germany), then passed through a 70 µm cell strainer and washed in RPMI. After centrifugation (300 x g, 10 min, 20 °C), red blood cells were lysed with 1 ml of lysis buffer (Red Blood Cell Lysing Buffer Hybri-Max™, Sigma) for 1 min. Splenocytes were then washed, suspended in RPMI-10 (RPMI supplemented with 10% FCS, 2 mM L-glutamine, 100 U penicillin, and 100 μg/ml streptomycin; all from GIBCO®, Thermo Fisher Scientific, Waltham, United States), and kept on ice until labeling.

Cell counts and viability were determined using a NovoCyte Flow Cytometer (Biosciences, San Diego, CA, USA) and 5 µg/ml of 7-amino-actinomycin D staining for 5 min in the dark (7-AAD, Life Technologies, Carlsbad, United States). After numeration, cells from PP, LP and spleen were centrifuged and suspended in FACS buffer containing FcR anti-CD16/anti-CD32 blocking reagent (clone 93, Biolegend; 1µg/10^6^ cells), and dispatched in 96-well V-bottom plates (10^6^ cells/well). Cells were first incubated with viability dye (LIVE/DEAD Fixable Dead Cell Stain Kit, 2 µg/mL, Invitrogen). After washing, different antibodies were used for immunophenotyping (from 0.125 to 2 µg of antibody/10^6^ cells, depending on the antibody used and pre-titration experiments). Antibodies belong to 3 panels designed to have an overview of all immune cell populations (ALL POP panel), and a deeper view of innate lymphoid cells (ILC Panel) and Th/Treg cells (Th Panel) subtypes. For cell surface staining, cell suspensions were incubated with the specific labeled antibodies for 30 min at 4 °C. After washing with PBS 1% FCS, stained cells were centrifuged (500 x g, 6 min, 4 °C) and suspended in CytoFix (BD Biosciences; ALL POP panel) or fixed and permeabilized (True-Nuclear Transcription Factor Buffer Set Kit, Biolegend; Th/Treg and ILC subsets analysis). In this later case, a new incubation with anti-CD16/CD32 antibodies was performed, followed by intracellular staining (Foxp3, RORγt, GATA-3, and Tbet) for 45 min at 4 °C. Stained cells were centrifuged (500 x g, 6 min, 4 °C), suspended in CytoFix and kept at +4 °C in the dark until flow cytometry data acquisition.

The anti-mouse antibodies used for extracellular and intracellular staining are provided in the corresponding dataset repository. Compensations were performed using beads (UltraComp eBeads Plus, Invitrogen) stained with the same antibodies. Unstained and mono-stained controls were performed by pooling all samples for each organ and each panel. Additionally, FMO controls were prepared for each cell marker. Data acquisition was performed on an Attune™ NxT Flow Cytometer (Thermo Scientific™, Waltham, MA, USA).

#### 4.2.2 Assessment of cytokines and total antibodies concentrations in plasma

After collection, blood was centrifuged (3500 x g, 20 min, +4 °C), and plasma was collected, aliquoted and kept at −20 °C until analysis. Cytokines (IFNγ, IL-5, IL-6, IL-10, IL-13, IL-17 and TNFα; kit form BioRad, Marne-la-Coquette, France) and total antibodies (IgG1, IgG2a, IgG2b, IgG3, IgM and IgA; kit from Millipore, Guyancourt, France) concentrations were measured in plasma diluted 1/4 and 1/25,000, respectively, following provider’s recommendations. Standard curves, positive quality controls and buffer only were included in each microtiter plate. Data were acquired on a Bio-Plex®200 suspension array system, after apparatus calibration and validation using commercial kits from Biorad. Concentrations were obtained using BioPlex Manager software.

### 4.3 Microbiota analysis by 16S rRNA amplicon sequencing

Total DNA was extracted from feces using the QIAamp power fecal DNA kit (Qiagen S.A.S., 51804, Hilden, Germany). DNA concentration was determined by spectrometry (NanoDrop, ThermoFisher Scienfic, Waltham, MA, US). 16S bacterial rRNA gene were amplified targeting the V3-V4 hyper-variable regions thanks to the following primers: F343 (CTTTCCCTACACGACGCTCTTCCGATCTTACGGRAGGCAGG) and R784 (GGAGTTCAGACGTGTGCTCTTCCGATCTTACCAGGGTATCTAATCCT). The PCRs were performed using 10 ng of fecal DNA, 0.5 µM of primers, 0.2 mM of dNTP, and 0.5 U of the DNA-free Taq-polymerase (MolTaq 16S DNA Polymerase, Molzym, Bremen, Germany). The PCR cycles were as follow: 1 cycle at 94 °C for 60 s, followed by 30 cycles at 94 °C for 60 s, 65 °C for 60 s, 72 °C for 60 s, and a final step at 72 °C for 10 min.

Single multiplexing was performed using a home-made 6-bp index, which was added to R784 during a second PCR with 12 cycles using forward primer (AATGATACGGCGACCACCGAGATCTACACTCTTTCCCTACACGAC) and reverse primer (CAAGCAGAAGACGGCATACGAGATGTGACTGGAGTTCAGACGTGT). Sequencing was performed at the @BRIDGe core facility (INRAE, Jouy-en-Josas, France; http://abridge.inrae.fr/) using Illumina Miseq technology. PCR products were purified and loaded onto the Illumina MiSeq cartridge according to the manufacturer’s instructions. Quality control of the sequence paired-end read merging process was assessed using four benchmark bacterial samples, which are routinely included as parallel controls by the sequencing facility . We also included negative controls that went through the same process as samples, from DNA extraction to sequencing. Raw 16S sequencing data can be uploaded to the Galaxy platform (https://galaxy.migale.inrae.fr/) [5,6] for analysis.

### 4.4 Metabolomic profiling by liquid chromatography coupled to high-resolution mass spectrometry (LC—HRMS)

#### 4.4.1 Sample preparation

Metabolites were extracted from plasma and feces samples following a pre-optimized protocol [7]. Proteins were precipitated by suspending samples in methanol/H2O (4:1, v/v). Feces samples were homogenized using CK14 ceramic beads and Precellys 24® apparatus (Bertin Technologies, Montigny-le-Bretonneux, France; 6500 x g, 4 °C, 330 s). Plasma samples were vortexed and incubated in an ultrasonic bath for 15 min. Then, plasma and feces homogenates were incubated on ice for 1.5 h. After centrifugation (20,000 x g, 4 °C, 15 min), supernatants (containing metabolites) were collected. Quality control (QC) was prepared by pooling equal volumes of all samples. Finally, all the samples and QC were dried under nitrogen (RotorVap LV-Biotage, 30 °C, 5 bars) and stored at −80 °C until data acquisition.

#### 4.4.2 LC-HRMS Analysis

The liquid chromatography coupled to high-resolution mass spectrometry (LC-HRMS) data acquisition was carried out using an Ultimate 3000 chromatographic system linked to a Q-Exactive mass spectrometer (both from Thermo Fisher Scientific, Courtaboeuf, France) equipped with an electrospray (ESI) source working in positive (ESI+) and negative (ESI-) ionization modes. To obtain a more comprehensive description of the metabolic landscape, LC was performed using two types of columns and ionization condition was adapted consequently: C18 (Hypersil GOLD C18 column, 1.9 m, 2.1 150 mm, Thermo Fisher Scientific; separation of apolar compounds; ESI+) and ZIC-pHILIC (Hydrophilic Interaction Liquid Chromatography; Sequant ZICpHILIC column, 5 m, 2.1 150 mm, Merck, Darmstadt, Germany; separation of polar compounds; ESI-).

The pellets were resuspended in 800 µL of ammonium carbonate (10 mM pH 10.5)/acetonitrile (ACN) (40:60, v/v) for HILIC separation, and H_2_O + 0.1% formic acid (FA)/ACN + 0.1% FA (95:5, v/v) for C18 separation. QC and diluted QC samples (1/2, 1/4 and 1/8) were prepared in the same solutions. One hundred microlitres of QC, diluted QC and biological samples were spiked with 5 µL of an external standard mixture, and 10 µL of the resulting samples were injected into the LC-HRMS system. Non-diluted QC samples were introduced every 5 biological samples for data normalization/standardization, whereas a dilution series of QC samples (1, 1/2, 1/4, and 1/8) were injected in triplicates at the beginning of the sequence to allow data filtration.

#### 4.4.3 Data treatment and annotation

Raw data (.raw files) were manually inspected using the Qual-browser module of Xcalibur (version 4.1, Thermo Fisher Scientific) and then converted to .mzXML format using MSconvert (“ProteoWizard”, version 3.0.21079). Data extraction was performed using the XCMS software package deployed on the open-source web-based W4M platform (https://workflow4metabolomics.org/) [8]. Filtration of the variables, as well as evaluation of quality metrics, were carried out using the phenomis R package (version 1.0.2) [9]. Features generated from XCMS were filtered according to the following criteria: (i) correlation between QC dilution factors and areas of chromatographic peaks (> 0.7), (ii) coefficient of variation of chromatographic peak areas of QC samples (< 30%), and (iii) ratio of chromatographic peak areas of biological to that of blank samples (> 3). Chromatographic peak areas of each variable were corrected for signal drift by fitting a locally weighted quadratic (LOESS) regression model to the QC values. Feature annotation was then performed along these later variables using an in-house spectral database based on chemical standards analyzed under the same analytical conditions [7,10]. To be annotated, features had to match accurately measured masses (± 10 ppm) and chromatographic retention times (±15 and 90 s for C18 and ZIC-pHILIC columns respectively). Each annotated peak was carefully checked using the Xcalibur Qual-browser module, taking into account the peak form, isotope pattern, and presence of the considered peak on at least six consecutive MS images. For the C18-ESI+ and HILIC-ESI- conditions, an intensity cut-off of 10,000 and 30,000 was used, respectively, to limit the existence of irrelevant peaks.

The appropriate signals were then subjected to MS/MS analysis to confirm their annotation allowing the highest level of confidence (level 1 or level 2, according to Metabolomic Standards Initiative) [11]. To get the best MS/MS spectra, four normalized collision energies (NCEs) were used (10, 20, 40, and 80%). It should be noted that some isomeric metabolites could not be resolved using the LC-HRMS method. As a result, some chromatographic peaks (for example, hexose) may correspond to more than one metabolite.

### 4.5 Transcriptomic analysis of jejunal tissue

Total RNA was purified from mid-jejunum tissue (5 mm cuts) using the RNeasy Midi & RNase-Free DNase Set (Qiagen S.A.S., 79254, Hilden, Germany), following the provider’s recommendations. The RNA concentration, 260/230 nm and 260/280 nm ratios were determined by spectrometry (Nanodrop). The RNA integrity number (RIN) was determined using a RNA 6000 Nano Chip and Agilent 2100 Bioanalyzer apparatus (Agilent Technologies, Santa Clara, CA). All samples had good RNA quantity and integrity (RIN ≥ 8).

These samples were hybridized on Mouse G3 gene expression v2 8 × 60 K microarrays (AMADID 074809, Agilent Technologies, Santa Clara, CA, US) at the @BRIDGe core facility (INRAE, Jouy-en-Josas, France; http://abridge.inrae.fr/). Cyanine-3 (Cy3)-labeled cRNA was prepared using 100 ng of total RNA (One-Color Low Input Quick Amp Labeling kit, Agilent Technologies). Specific activities and cRNA yields were determined (Nanodrop 2000, Thermo Fisher Scientific). For each sample, 600 ng of Cy3-labeled cRNA (specific activity > 9.0 pmol Cy3/µg of cRNA) was fragmented at 60 °C for 30 min and used for hybridization (17-h at 65 °C under rotation in a hybridization oven, Agilent Technologies). After hybridization, microarrays were washed and the slides scanned (G2565CA Scanner System, Agilent Technologies; resolution of 3 µm and dynamic range of 20 bits). The resulting .tiff images were analyzed using Feature Extraction Software version 12.0.3.2 (Agilent Technologies, GE1_1200_Jun14 protocol).

## Limitations

Comparison of the results with that obtained by other researchers may be jeopardized due to variability resulting from husbandry and handling conditions, in addition to experimental conditions such as strain, sex, age, diet, sample collection, and analytical procedures and apparatus.

## Ethics Statement

The animal study was reviewed by our local ethical committee and approved by the French Minister (authorization #28048-A20_049).

## CRediT Author Statement

**Mohammad Issa**: Investigation, Data Curation, Formal analysis, Visualization, Writing - Original Draft. **Chloé Michaudel:** Data Curation, Validation, Writing - Original draft. **Blanche Guillon**: Investigation, Data curation. **Marine Guinot**: Investigation. **Marta Grauso**: Investigation. **Jérôme Lecardonnel**: Investigation. **Luc Jouneau** : Investigation, data curation. **Céline Chapuis** : Investigation. **Hervé Bernard** : Investigation. **Stéphane Hazebrouck** : Investigation. **Florence Castelli**: Ressources. **François Fenaille**: Ressources. **Eric Gaultier**: Investigation. **Gilles Rivière**: Supervision. **Eric Houdeau**: Conceptualization, Supervision. **Karine Adel-Patient**: Project administration, Supervision, Data Curation, Validation, Writing - Original Draft, Funding acquisition.

## Data Availability

data.gouv.fr/dataverse/inraeDatasets of plasma and fecal metabolites in young BALB/cAnNRj female and male mice exposed or not to food additive E171 from preconception(Original data)
data.gouv.fr/dataverse/inraeDatasets of fecal microbiota in young BALB/cAnRj female and male mice exposed or not to food additive E171 from preconception(Original data)
data.gouv.fr/dataverse/inraeDatasets of jejunal transcriptome in young BALB/cAnRj female and male mice exposed or not to food additive E171 from preconception(Original data)
data.gouv.fr/dataverse/inraeDatasets of small intestine and systemic immune components in BALB/cAnRj young female and male mice exposed or not to food additive E171 from preconception(Original data)
